# Use the brain: complementary methods to analyse the effects of motivational music

**DOI:** 10.3389/fnhum.2015.00508

**Published:** 2015-09-16

**Authors:** Marcelo Bigliassi

**Affiliations:** Department of Life Sciences, Brunel University LondonUxbridge, UK

**Keywords:** music, motivation, brain, heart, exercise

## The motivational qualities of music

Music-related interventions have been extensively used in the field of sport and exercise as a strategy to ameliorate the effects of fatigue-related symptoms and enhance exercise performance (for review, see Karageorghis and Priest, 2012a,b). Recent evidence indicates that music regulates brain activity (Bishop et al., 2014) with consequent effects on psychophysiological responses and work output (Terry et al., 2012). In order to define whether a piece of music is motivational or *oudeterous* (i.e., motivationally neutral) psychological instruments such as the Brunel Music Rating Inventory-3 (BMRI-3; Karageorghis and Terry, 2011) have been developed on the premise that the musical components define the motivational qualities of music. Karageorghis et al. (2006) suggested that musical components such as rhythm and melody have considerable influence upon psychophysiological responses that occur during exercise. In such application, the exerciser rates the magnitude by which the elements of music influence the physical task. Each piece of music receives a score based on the exerciser's/athlete's opinion; thus, the score is used as an index of how motivational a piece of music is (e.g., Hutchinson and Karageorghis, 2013). Despite the influence of the elements of music on bodily responses that occur during exercise, moderate-to-high intensity bouts of physical activity are theorized to force attentional focus to associative thoughts, meaning that subcomponents of the auditory stimulus are naturally dismissed (see the Attenuation Theory, Treisman, 1964; see Gabana et al., 2015, for a practical example).

## Attention, exertion, and sensory information

Physical tasks performed at moderate-to-high intensity induce symptoms of fatigue with consequent detrimental effects on exercise performance (Gandevia, 2001). The brain has limited capacity to process sensory signals (Rejeski, 1985), meaning that afferent feedback (Pollak et al., 2014) and corollary signals (De Morree et al., 2012, 2014; Bigliassi, 2015) reallocate an individual's attentional focus to the sensations of fatigue (Hutchinson and Tenenbaum, 2007). In this case, the effects of music decrease over time due to the increasing symptoms of fatigue (Bigliassi et al., 2015b). If intensity of the exercise increases, the human brain naturally reallocates the attentional focus to the signals considered crucial for the task at hand (Treisman, 1964). Perceived exertion is hypothesized to modulate attentional focus to associative thoughts such as internal sensory cues and task-related information (e.g., Razon et al., 2009; Hutchinson et al., 2011). Based on this assumption, moderate-to-high-intensity exercise imposes considerable difficulties for the human brain, which means that severe physical exertion partially limits the processing of task-unrelated information such as music.

## The aim of this communication

The aim of this article is to highlight the use of complementary techniques to explore the motivational effects of music and its application for future research in the field of sport and exercise. The rationale of the present piece of work lies in the fact that exercisers can only focus on the general message of music during moderate-to-high intensity bouts of physical activity, because internal sensory cues force attentional focus to associative thoughts such as internal processes (e.g., muscle discomfort and heart rate) and task-related indices (e.g., the teleoanticipation mechanism; Wittekind et al., 2011).

## Neurophysiological and psychological connections

The mechanisms by which motivational music influences psychophysiological responses are hitherto under-researched (Karageorghis and Priest, 2012a). The brain is theorized to process music differently during exercise due to the effects of fatigue (Rejeski, 1985; Hutchinson and Karageorghis, 2013). Jones et al. (2014) suggested that even high-intensity exercise can feel more pleasurable under the influence of music, meaning that subcortical structures such as the anterior cingulate cortex and amygdala are possibly responsible for processing music at this stage (Chanda and Levitin, 2013). In this case, little processing is necessary for music to influence an individual's affective state. The results of Jones et al. (2014) are partially supported by the notion that a pleasant stimulus not only activate one sensory pathway, but also blocks the activity of opposite regions of the brain (Hernández-Peón et al., 1961). Theoretically, motivational music represents a “barrier” against exertional responses. However, the increasing symptoms of fatigue are generally stronger than the effects of music, which means that it is only “a matter of time” until fatigue-related symptoms overcome the protective effects of music (Rejeski, 1985; Bigliassi, 2015).

Compelling evidence indicates that cascade reactions from brain activity to visceral changes (e.g., cardiac responses) underlie the effects of music (see the neurohumoral pathway proposed by Conrad et al., 2007). The prefrontal cortex (PFC) appears to be highly responsive to auditory stimulation (Green et al., 2012) and a series of studies have been conducted in order to unearth the mechanisms that underlie the psychophysiological effects of music (Bigliassi et al., 2015a,b). The PFC was examined through the use of functional near-infrared spectroscopy (fNIRS), which is an innovative technique applied to the field of psychophysiology (e.g., Moghimi et al., 2012). Findings indicate that stimulative (highly arousing stimulus) music has considerable influence on the oxygenation of the PFC with subsequent effects on the activity of the sympathovagal balance (time domain analysis). Additionally, brain assessment techniques have been applied before exercise as a means to equalize the motivational effects of music on psychophysiological variables (Bigliassi et al., 2015b). In such application, even extremely different pieces of music increased the activity of the PFC in a similar fashion. This result is supported by the notion that non-musicians are theorized to focus on the general message carried by music, while musicians are naturally concerned about the constituent components of music such as pitch and rhythm (Levitin, 2008).

## Why don't we use the brain?

Music has the “power” to elicit affective responses, control arousal, and evoke long-term memories (Jäncke, 2008). The PFC appears to be highly responsive to this stimulus (Green et al., 2012), but other brain regions are also sensitive to music such as the auditory cortex, amygdala and hippocampus (Koelsch, 2010; Koelsch and Skouras, 2014). This article has demonstrated that cortical activity, visceral changes, and psychological responses could be used in future research to standardize the effects of motivational music. Methods to analyse the brain (e.g., electroencephalography and fNIRS) and psychophysiological techniques (e.g., heart rate variability and galvanic skin conductance) should be applied during the initial stage of the experiment in order to explore and monitor the effects of music. In such application, even different pieces of music are hypothesized to elicit similar psychophysiological reactions, due to the fact that music is processed according to idiosyncratic patterns of response (see the Five-Factor Model proposed by Rentfrow et al., 2011). Accordingly, researchers are encouraged to use complementary methods as a means by which to standardize the “dosage” of music based on the psychological responses and bodily reactions that such stimulus induces. The motivational impact of music can be identified at rest through the use of psychophysiological indices. The same tracks can be subsequently applied during exercise-related situations if they are similarly motivational.

### Conflict of interest statement

The author declares that the research was conducted in the absence of any commercial or financial relationships that could be construed as a potential conflict of interest.
